# Highlighting Recent Progress in Fiber Energy Harvesters: From Working Principles to Future Perspectives

**DOI:** 10.1002/advs.202505784

**Published:** 2026-03-10

**Authors:** Hanhwi Jang, Junseong Ahn, Yongrok Jeong, Sohee Jeon, Soon Hyoung Hwang, Min‐Wook Oh, Jun‐Ho Jeong, Inkyu Park, Yeon Sik Jung

**Affiliations:** ^1^ Department of Materials Science and Engineering Korea Advanced Institute of Science and Technology (KAIST) Daejeon Republic of Korea; ^2^ Department of Control and Instrumentation Engineering Korea University Sejong Republic of Korea; ^3^ School of Mechanical Engineering Kyungpook National University Daegu Republic of Korea; ^4^ Nano Lithography and Manufacturing Research Center Korea Institute of Machinery and Materials Daejeon Republic of Korea; ^5^ College of Pharmacy Chung‐Ang University Seoul Republic of Korea; ^6^ Department of Materials Science and Engineering Hanbat National University Daejeon Republic of Korea; ^7^ College of Medicine Chung‐Ang University Seoul Republic of Korea; ^8^ Department of Mechanical Engineering Korea Advanced Institute of Science and Technology (KAIST) Daejeon Republic of Korea

**Keywords:** energy harvesters, fabrication, fibers, flexible, wearable

## Abstract

As electronics proliferate through the rise of the internet of things (IoT) and artificial intelligence (AI), the need for sustainable, decentralized power source is growing. Energy harvesting—converting ambient sources such as vibration, heat, or electromagnetic waves into electricity—offers a promising solution for powering distributed, low‐power, or wearable electronic systems. However, the practical deployment of most energy harvesters has been significantly limited by the processability issues associated with the inherent brittleness of conventional materials. In contrast, fiber‐based energy harvesters offer superior flexibility and stretchability due to their intrinsic deformability and multidirectional bending capabilities, presenting a compelling alternative to conventional energy harvesters. This review systematically summarizes the fabrication processes and performance characteristics of fiber energy harvesters, categorizing them by the origin of the energy source—mechanical, optical, and thermal. In particular, various design considerations based on the working principles of fiber energy harvesters are retrospectively analyzed to provide guidelines for developing next‐generation fiber energy harvesters. Additionally, current challenges and future research directions are discussed, highlighting the potential of fiber‐based platforms to enable next‐generation wearable electronics.

## Graphical abstract

Fiber energy harvesters offer unprecedented flexibility and a unique capacity for integration into commercial textiles, overcoming the limitations of bulky and rigid conventional devices. This review summarizes recent advances in fiber‐based energy harvesting and provides strategic outlooks to accelerate technological progress in the field.

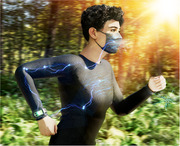

## 1 Introduction

The recent advances in the internet of things (IoT), artificial intelligence (AI), and modern electronics have significantly transformed our daily lives, leading to an exponential increase in the density of electronic devices [1]. Consequently, the necessity of powering decentralized electronic devices through distributed energy resources has been persistently growing [2, 3, 4]. In response, the concept of energy harvesting–which involves converting optical, mechanical, and thermal energy into electricity that would otherwise be wasted–has gained considerable attention as a sustainable strategy to address the global energy crisis [5, 6, 7].

Traditional energy harvesters have long aimed to improve their energy conversion efficiency through various strategies, including doping and alloying, nanostructuring, heat treatment, and passivation [8, 9, 10, 11, 12, 13]. As a result, modern energy harvesters have evolved beyond merely sending signals for voltmeters; they can now activate light‐emitting diodes (LEDs) [14], power chemical or physical sensors [15], and even charge batteries for practical applications [16, 17]. These demonstrations show that the energy harvesters may be capable of supplying electrical power to individual electronic devices, thereby promoting the digitalization of everyday life by reducing the carbon footprint.

Given the diverse morphologies and operating environments of various energy sources, flexibility and stretchability are critical properties for advanced wearable electronics [18, 19]. However, one aspect that has been largely overlooked is the processability of energy harvesters; most designs have paid little attention to the mechanical properties essential for flexible and stretchable operation [20, 21]. From a materials perspective, functional energy harvesting materials are primarily composed of brittle ceramics or polymers with inherently poor mechanical properties [22, 23, 24]. Consequently, when used in bulk form, these materials are prone to mechanical failure under repeated deformation or vibration, which significantly limits the practical applicability of bulk energy harvesters [25, 26, 27, 28]. Although thin‐film energy harvesters exhibit moderate mechanical properties compared to their bulk counterparts, they are limited to out‐of‐plane bending, which results in a lack of the flexibility and stretchability required for portable devices such as wearable electronics.

**FIGURE 1:**
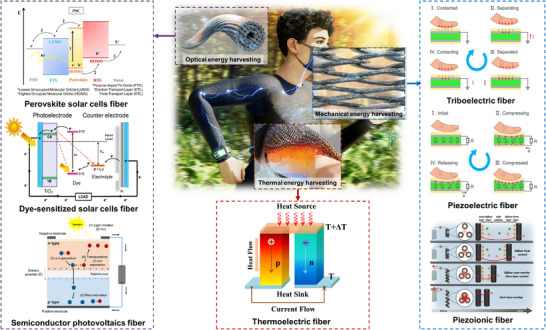
Schematic illustration of various fiber energy harvesting devices integrated into wearable devices. Thermal, mechanical, and optical energy are converted into electricity to power a smartwatch using body heat, breathing, and sunlight, respectively. Schematics for optical energy harvesting were reproduced with permission from Ref. [49], Copyright 2021, The Authors, MDPI; and Ref. [50], Copyright 2021, Elsevier Ltd. Mechanical energy harvesting schematics were reproduced from Ref. [51] under the terms of the CC‐BY 4.0 license, Copyright 2022, Springer Nature; and Ref. [52] with permission, Copyright 2024, Wiley‐VCH GmbH. The thermal energy schematic was reproduced with permission from Ref. [53], Copyright 2023, The Authors, MDPI.

Compared to thin films, fiber energy harvesters offer more degrees of freedom in the bending direction, leading to excellent tensile, bending, and shear properties, and hold great promise for wearable applications [29, 30]. They can withstand repeated deformations–such as stretching, twisting, bending, and shearing–while maintaining structural integrity. Moreover, the inherent deformability of highly soft fibers allows them to conform to the curved and uneven surfaces such as human body. Figure 1 schematically illustrates how fibers can harvest mechanical, thermal, and optical energy to generate electrical power for future wearable electronics. For example, mechanical vibrations of functional fibers in an energy‐harvesting mask can generate electricity via triboelectric nanogenerators. Some representative triboelectric nanogenerators in wearable and flexible applications are well highlighted in recent review articles [31, 32]. Similarly, a temperature gradient between the human body and the ambient environment creates an electric potential through the thermoelectric effect. Outdoor light during the daytime can be efficiently harvested using fiber photovoltaic devices integrated into garments. Together, this electrical power can supply enough energy to operate other devices, such as a smartwatch or health monitoring devices. Several examples of fiber energy harvesters are summarized in Table 1 and will be discussed in the following sections.

**TABLE 1:** Summary and key characteristics of fabrication processes for representative fiber energy harvesters.

Classification	Materials	Process	Characteristics	Refs.
Bottom–up (electrospinning)	Polymers	Electrospinning of polymer solution and twisting of electrospun fibers as yarn shape	Highly beneficial for its scalability and efficiency, making it ideal for mass production	[33]
Bottom–up (chemical vapor deposition)	Carbon nanotube and graphene	Vertically growing carbon‐based materials and twisting of aligned fibers as yarn shape	Well‐suited for producing mechanically stable, highly conductive, and lightweight carbon‐based fibers	[34]
Bottom–up (electron beam deposition)	Inorganic materials (e.g., metals and ceramics)	Electron beam deposition of inorganic elements on nanopatterned mold followed by reactive ion etching, and yarn twisting	Enables inorganic material fabrication, making it suitable for high‐performance transducers in energy harvesting, storage, and sensing applications	[35, 36, 37]
Top–down	All‐ink‐compatible materials	Coating of commercially available fibers or yarns by dip‐coating, spray coating, etc.	Compatible with various materials, offering high versatility, but limited by the inability to produce single‐material fibers	[38, 39]

In this review, we highlight the advantages of fiber energy harvesters relative to the conventional energy harvesters, overcoming the intrinsic limitations of their bulk counterpart. We first summarize the characteristics of fiber energy harvesters according to their fabrication processes (Section 2), followed by a detailed classification of applications based on mechanical, optical, and thermal energy sources. Finally, we discuss current challenges and envision the future outlook of the fiber energy harvesters as a basis for future research directions (Section 4) (Table 2).

**TABLE 2:** Representative examples of fibrous mechanical, thermal, and optical energy harvesters and their applications.

Energy source	Classification	Description	Output characteristics		Applications	References
Mechanical	Piezoelectric	Carbon black (CB) / PVDF core fiber with melt‐spun PVDF sheath	0.8 mW/m^2^ for piezoelectric fabric		Piezoelectric shoulder strap	[40]
	Triboelectric	Conductive silver core fiber with electro‐spun precursor solutions composed of PVDF and PAN	40.8 V, 0.705 µA/cm^2^, and 9.513 nC/cm^2^ with 2.5 Hz mechanical drive for single yarn		Fingertip contact sensor, caterpillar crawling identification, and smart textile for biomechanical sensing	[41]
	Piezoionic	Twisting of cone‐spun, highly oriented CNTs, and there multiplying	17.4% of tensile energy harvesting efficiency, 22.4% of torsional energy harvesting efficiency		Flow velocity sensor and arm bending energy harvester	[42]
Thermal	Inorganic/polymer composites	3D‐extruded Bi_2_Te_3_/ polylactide‐*co‐*glycolide composite	Open circuit voltage (mV)	8 mV at a temperature difference of 10 K	Demonstrated parallel and transverse module with compression test	[43]
		Self‐standing all‐inorganic Bi_2_Te_3_ yarn		67.4 mV using a heat gun	Woven in the life jacket and garments for wearable electronics	[35]
	Core‐and‐cladding glass fibers	Recrystallized SnSe core glass fiber		30 mV at a temperature difference of 10 K	Woven into fabrics with breathability and flexibility	[44]
	Carbon nanotubes	Selectively doped CNT yarns		250 mV at a temperature difference of 1.5 K	Powering LEDs and electrochromic devices while coiled at the wrist	[45]
Optical	Dye‐sensitized solar cells	TiO_2_‐integrated aligned carbon nanotube	Power conversion efficiency	25.53% under 1500 lux illumination (indoor energy harvesting)	Self‐charging power textile for a wearable healthcare system	[46]
	Semiconductor photovoltaics	Diced crystalline silicon cells‐integrated flexible circuit strip		11% under air mass 1.5G illumination (outdoor energy harvesting)	Woven into textile swatches and driving light‐emitting diode	[47]
	Perovskite solar cells	Perovskite materials‐coated spring‐shaped Ti wires		5.22% under air mass 1.5G illumination (indoor and outdoor energy harvesting)	Elastic textile	[48]

## 2 Fabrication Methods of Fiber Energy Harvesters

Technically, a ‘fiber’ refers to a single continuous filament or the basic discrete unit of a material, whereas a ‘yarn’ denotes an assembly of fibers twisted or bundled together to form a continuous strand suitable for textile processing. Although these two terms are often used interchangeably in literature, the distinction is crucial for energy harvesting applications; given the limited energy‐harvesting capacity of a single fiber, the yarn typically serves as the practical structural motif for delivering functional power. With this structural distinction in mind, the fabrication of fibers or yarns energy harvesters generally follows two primary approaches: (1) bottom–up (fibers to yarn) methods, where nanomaterials are synthesized and assembled into fibrous structures, and (2) top–down (coating of commercially available yarns or fibers) methods, where functional materials are deposited onto pre‐existing fiber substrates. Furthermore, (3) additional processing methods can be applied, such as controlling the twisting configuration, structural integration of multiple fibers or yarns, and creating unique structures such as core–spun structures. The final properties of the fabricated fiber, including material composition, twisting direction, and morphological structure, depend on the processing methods chosen at each step. These parameters must be optimized based on the intended application to ensure the required mechanical, electrical, or optical characteristics.

### 2.1 Bottom–Up (Fibers to Yarn) Methods

Bottom–up fabrication methods synthesize fibers from fundamental building blocks such as nanowires or nanoparticles before assembling them into yarns. Representative techniques include electrospinning, chemical vapor deposition (CVD), and electron beam deposition (EBD). Electrospinning enables scalable production of stretchable polymer fibers, while CVD offers direct growth of conductive carbon nanomaterials with excellent mechanical stability. EBD provides precise control over fully inorganic, multilayered nanowires, expanding material options for high‐performance functional fibers. We now discuss the characteristic features and advantages of each technique in detail.

Electrospinning is one of the most commonly employed techniques for producing polymer‐based fibers [33]. It operates based on the principle of electrostatic force‐driven jet elongation, where a high‐voltage electric field is applied to a polymer solution or melt, generating a charged fluid jet that extends and solidifies into ultrafine fibers due to electrostatic repulsion and surface tension reduction. The key parameters governing the process include applied voltage, current, and nozzle diameter, which directly influence the resulting nanowire dimensions. Electrospinning is particularly advantageous for its low‐cost, high‐throughput nature, making it suitable for large‐scale production. Furthermore, the compatibility of this technique with polymer materials allows for the fabrication of highly stretchable fibers, which can be further utilized as templates for additional functionalization via nanoparticle or molecular coatings. Additionally, core‐spun structured fibers can be fabricated using dual‐nozzle electrospinning, enabling the incorporation of multiple material functionalities within a single fiber. Despite these advantages, electrospinning has several limitations. It is primarily restricted to specific polymers and solvent systems, and the resulting fibers often exhibit lower mechanical strength and electrical conductivity compared to carbon‐based or inorganic fibers. Furthermore, the process is highly sensitive to environmental factors such as humidity and temperature, which can lead to poor reproducibility and non‐uniform fiber morphology if not strictly controlled.

Another well‐established approach is CVD, a technique widely utilized for growing carbon‐based nanomaterials such as carbon nanotubes (CNTs) and graphene yarns. CVD involves the decomposition of gaseous precursors under controlled temperature and pressure, leading to the formation of nanowires on a designated substrate [34]. The growth parameters, including chamber temperature, precursor concentration, gas flow rate, and reaction time, determine the structural characteristics and properties of the synthesized nanowires. This method is particularly effective for producing highly conductive materials with exceptional mechanical and chemical stability. Although early implementations of CVD‐based nanowire growth faced challenges related to efficiency and reproducibility, recent advancements in process optimization have enabled its successful integration into commercial applications. CNT and graphene‐based fibers have demonstrated significant potential in applications such as flexible electronics, chemical sensors, and high‐performance energy storage systems. However, the primary drawbacks of CVD include its high energy consumption necessitated by high operating temperatures and the requirement for high‐vacuum environments. The use of hazardous gaseous precursors and the high cost of equipment pose significant barriers to cost‐effectiveness. Additionally, the growth rate is relatively slow, making it less efficient for rapid, large‐scale production compared to electrospinning.

The third method for fabricating fibers or yarns is EBD, a technique that has only recently been reported as of 2024 [36, 37]. This method enables the fabrication of fully inorganic nanowires with precise compositional control and scalability. The process begins with the preparation of a nanoimprinted mold, in which a patterned mold with nanoscale grooves or lines is created to define the shape and alignment of the nanowires. The target inorganic material is then deposited onto this mold via EBD, allowing for precise layer thickness control at the nanometer scale. Subsequently, plasma‐assisted reactive ion etching is employed to undercut the mold, effectively suspending the deposited nanowires in a freestanding configuration. This critical step minimizes adhesion between the mold and the nanowires, facilitating their delamination and subsequent twisting into fiber structures. One of the major advantages of this technique is its versatility in material selection. By altering the source materials in the e‐beam evaporator, a variety of inorganic materials can be incorporated into nanowires, including Au, Pd, Ni, Al, Pt, WO_3_, SnO_2_, NiO, In_2_O_3_, CuO, Bi_2_Te_3_, and BiSe. Furthermore, layer‐by‐layer deposition enables the fabrication of multilayered nanowires. Given that most high‐performance electronic devices rely on inorganic materials, this technique holds significant potential for applications in the field of functional fibers such as flexible sensors, energy harvesting devices, and advanced optoelectronic systems. Despite its versatility and precision in material selection, EBD‐based fabrication faces challenges related to process complexity. It involves multiple steps, including nanoimprinting and reactive ion etching, which require expensive cleanroom facilities. Moreover, current implementations are limited to centimeter‐scale production, and the throughput is significantly lower than that of continuous electrospinning or CVD processes. The necessity of a high‐vacuum environment for electron beam evaporation further adds to the operational cost.

In summary, comparing these bottom–up techniques, electrospinning offers the highest scalability and cost‐efficiency for polymer‐based fibers but lacks material diversity. CVD provides superior electrical and mechanical properties for carbon nanomaterials but at the cost of high energy and hazardous precursors. EBD enables the most precise control over inorganic multi‐layers but remains the most complex and expensive method with limited scalability at present.

### 2.2 Top–Down (Coating of Commercially Available Fibers or Yarns) Methods

Top–down fabrication methods involve functionalizing pre‐existing fibers or yarns, such as commercially available cotton, polyester, and silk, by depositing active materials onto their surfaces. A representative technique is dip coating [38, 39], in which fibers are immersed in a solution containing nanomaterials and withdrawn at a controlled rate to achieve a uniform coating. Alternative coating methods, such as spray coating and inkjet coating [54, 55], allow for precise deposition of functional materials. This approach facilitates the integration of nanomaterials into conventional fiber substrates, offering scalability and compatibility with various materials. To ensure effective performance, these substrates must possess high mechanical flexibility, excellent surface wettability for coating adhesion, and thermal/chemical stability to withstand the functionalization process. However, top–down methods have distinct disadvantages compared to bottom–up approaches. A major challenge is the poor interfacial adhesion between the active coating layer and the fiber substrate, which can lead to delamination during repeated mechanical deformation. Additionally, achieving a perfectly uniform coating thickness along the entire length of the fiber is difficult, often resulting in inconsistent device performance. Unlike bottom–up methods, this approach is also limited by the properties of the underlying substrate, which may restrict the overall flexibility or thermal stability of the final energy harvester.

While bottom–up methods allow for the creation of high‐purity, single‐material fibers with intrinsic functionalities, top–down methods (e.g., dip coating) remain the most practical for rapid integration with existing textile infrastructures, despite their limitations in durability and material homogeneity.

### 2.3 Additional Processing Methods

Beyond the fundamental fabrication methods, additional processes can be applied to enhance or introduce new functionalities. These optional processes can be categorized into three types: single plying (twisting), multiplying, and additional plying techniques [56, 57].

**FIGURE 2:**
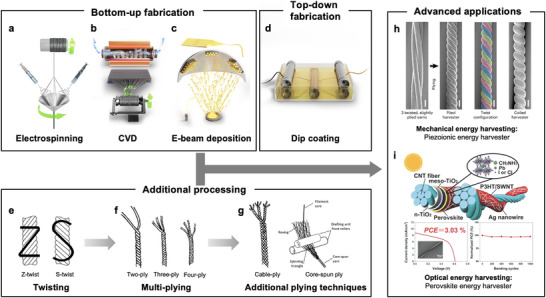
Fabrication methods for fiber energy harvesters. Representative bottom–up fabrication methods: (a) electrospinning, (b) chemical vapor deposition, and (c) electron beam deposition. Representative top–down fabrication method: (d) dip coating. (e) Detailed twisting (single‐plying) method of multiple fibers: Z‐twist and S‐twist. Reproduced with permission [56], Copyright 2020 Elsevier. (f) Examples of the multiplying method. Reproduced under the terms of the CC‐BY‐4.0 license [57], Copyright 2016 Science PG. (g) Additional plying techniques: cable‐ply and core‐spun ply. Reproduced under the terms of the CC‐BY‐4.0 license [57], Copyright 2016 Science PG. (h) Example of utilizing the multiplying method to enhance system functionality. Reproduced under permission [42], Copyright 2023 Springer Nature. (i) Example of utilizing the multiplication of heterogeneous yarns to fabricate a complex system within a yarn structure. Reproduced with permission [59], Copyright 2015 Wiley‐VCH GmbH.

In the single‐plying process, also known as twisting, multiple fabricated fibers are gathered into a single yarn. This twisting can follow two directionalities: Z‐twist and S‐twist (Figure 2e). In general, the physical properties of the yarn do not significantly change depending on the twist type, while it determines the subsequent twist orientations in multiplying and additional plying processes.

Multiplying involves twisting multiple strands of single‐plied yarn together (Figure 2f). Typically, the twisting direction for plying is the opposite for that of a single yarn. For example, a three‐plied yarn can be formed by Z‐twisting three S‐twisted yarns or by S‐twisting three Z‐twisted yarns. The reverse‐direction twisting during the multiplying process adjusts the overall twist level of the final yarn, ensuring a more stable structure. Generally, as the number of plied yarns increases, the final yarn's cross‐section becomes thicker, making it stronger. Additionally, multiplying improves the durability, elongation capacity, and stability of the yarn. By varying the fiber types used in the plying process, it is also possible to engineer the final yarn's properties by combining different fiber materials [58].

Multiplied yarns can undergo further plying methods, such as cable‐plying and core‐spun plying (Figure 2g). Cable‐plying is an additional multiplying process that further enhances the stability and durability of the overall structure. Core‐spun plying, on the other hand, involves twisting fibers around a core filament, resulting in a unique yarn structure.

These additional techniques have been widely utilized in numerous research. For example, Zhang et al. maximized the piezoionic harvesting of mechanical energy using a three‐plied CNT yarn, termed twistrons (Figure 2h) [42]. Piezoionic energy harvesting generates energy based on the change in supercapacitance between the external ionic liquid and the yarn, which varies depending on the actual contact area of the liquid with the yarn. In their study, they aligned the twisting direction of the CNT fibers with the plying direction of the yarn to form a three‐plied twistron, thereby maximizing the effective area variation and achieving high mechanical energy harvesting efficiency. Additionally, Ru et al. successfully fabricated a perovskite energy harvester by multiplying heterogeneous yarns (Figure 2i) [59]. By plying a CNT yarn coated with a perovskite solar cell (PSC) together with a conventional CNT yarn used as the cathode, they successfully integrated necessary components of a solar cell into a single yarn.

## 3 Applications in Energy Harvesting

To transition from individual functional fibers to practical energy harvesting systems, these components must be integrated into macroscopic structures. Following the fabrication of functional yarns via the methods described in Section 2, they are typically integrated into textile platforms using various techniques such as weaving, knitting, or embroidery, depending on the desired mechanical properties and breathability. Detailed examples of these integrated applications are provided in Sections 3.1, 3.2, and 3.3.

### 3.1 Mechanical Energy

Fiber mechanical energy harvester holds significant potential in bio‐energy harvesting, especially utilized in wearable applications [29, 60, 61]. In detail, the mechanical deformation such as compression, contact, and stretching of the clothing, caused by the movement of the human body, can be converted into electrical energy. In this section, we explore three representative strategies for fiber‐based mechanical energy harvesting: piezoelectric, triboelectric, and dielectric or electrochemical processes.

**FIGURE 3:**
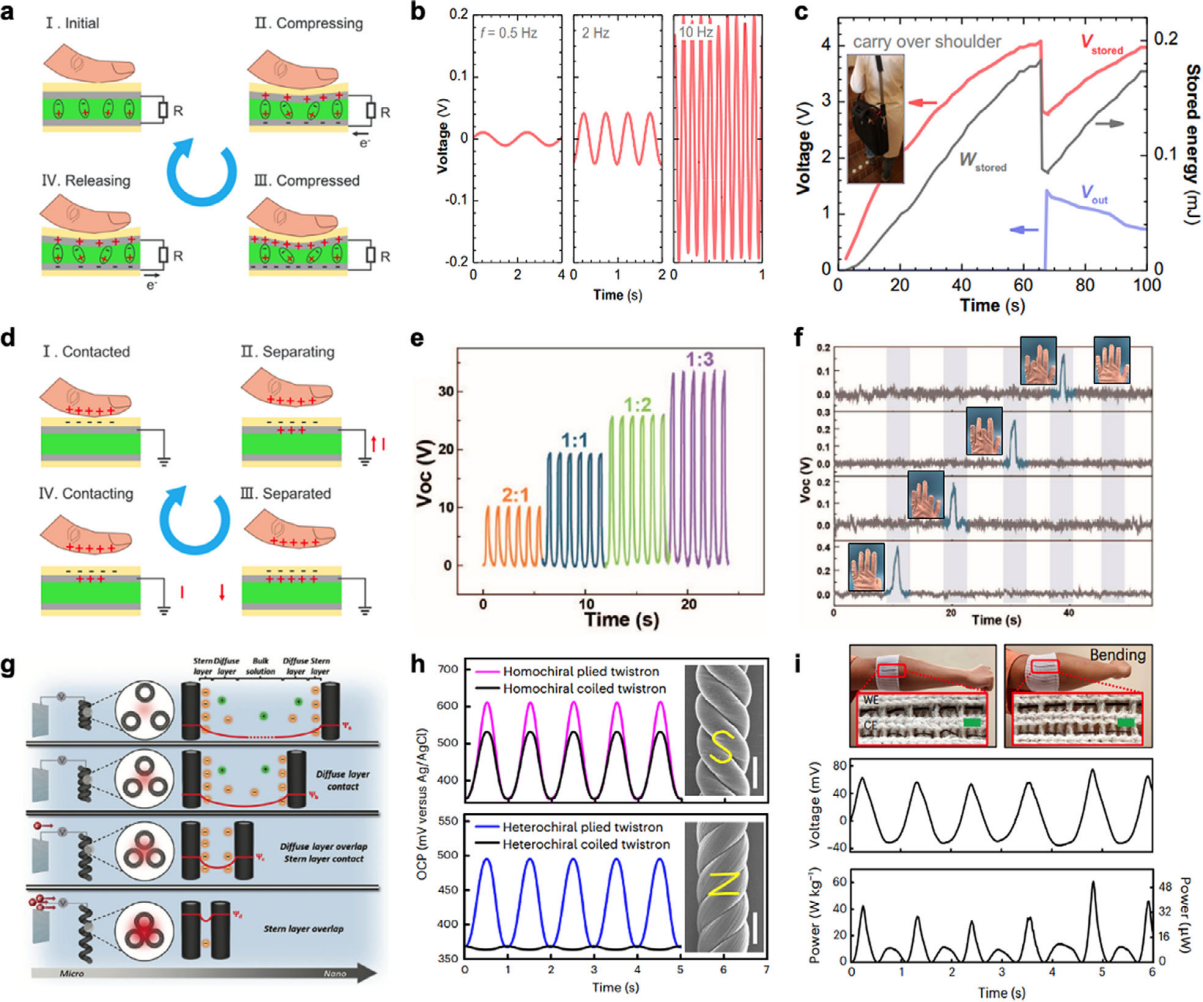
Fiber‐based mechanical energy harvesters. (a) Principle of piezoelectric energy harvesters. Reproduced under the terms of the CC‐BY‐4.0 license [51], Copyright 2022 Springer Nature. (b) Example output graph of fiber‐shaped piezoelectric energy harvesters. Reproduced under the terms of the CC‐BY‐4.0 license [40], Copyright 2018 Springer Nature. (c) Applications of piezoelectric energy harvesters in carry over shoulder bag. Reproduced under the terms of the CC‐BY‐4.0 license [40], Copyright 2018 Springer Nature. (d) Principle of triboelectric energy harvesters. Reproduced under the terms of the CC‐BY‐4.0 license [51], Copyright 2022 Springer Nature. (e) Example output graph of fiber triboelectric energy harvesters. Reproduced with permission [41], Copyright 2018 American Chemical Society. (f) Application of triboelectric energy harvesters in gloves for a fingertip contact monitoring system. Reproduced with permission [41], Copyright 2018 American Chemical Society. (g) Principle of piezoionic energy harvesters. Reproduced with permission [52], Copyright 2024 Wiley‐VCH GmbH. (h) Example output graph of fiber piezoionic energy harvester depending on the plying method. Reproduced with permission [42], Copyright 2023 Springer Nature. (i) Application of fiber piezoionic energy harvester in a bioenergy harvesting device. Reproduced with permission [42], Copyright 2023 Springer Nature.

Piezoelectric materials can convert mechanical energy by compression into electrical energy (Figure 3a). Specifically, the applied pressure causes the displacement of ions within their atomic structure, and this imbalance generates a voltage difference. This voltage difference induces an electrical current, which can then be stored in specific energy harvesting devices such as batteries and capacitors. However, conventional high‐performance piezoelectric materials are not only brittle but also require a thermal annealing process, making it difficult to fabricate them in flexible fiber form. Therefore, for flexible fiber‐based piezoelectric energy harvesters, polymer‐based piezoelectric energy harvesters, primarily using poly(vinylidene fluoride) (PVDF), have been actively developed [40, 62, 63, 64, 65]. For example, Lund et al. produced PVDF fibers from melt‐spun PVDF microfibers, evaluated their piezoelectric properties (Figure 3b), and weaved them into create bag straps, developing a wearable mechanical energy harvester (Figure 3c) [40]. Flexibility can be granted from not only polymer, but also nanostructured materials. Li et al. demonstrated the feasibility of fabricating piezoelectric fiber energy harvesters by growing ZnO nanowires on carbon fibers and utilizing the pressure‐induced deformation of the nanowires to generate electricity [66].

Triboelectric materials can generate electrical energy through contact (Figure 3d). In detail, when two materials come into contact, charge migration occurs at the contact boundary. Upon detachment, the movement of these charges induces a charge flow, leading to the production of electrical energy [67, 68, 69]. The greater the difference between materials in the triboelectric series, the more significant the charge migration at the contact interface [70]. Triboelectric fiber energy harvesters have been studied in two major structural configurations. The first type features a core–shell structure, with research conducted on material pairs such as poly(tetrafluoroethene)/poly(dimethyl siloxane) (PTFE/PDMS) [71], nylon/silicone [72], and silicone/Cu [73]. The second type consists of a single fiber with an embedded electrode for the charge transfer, enabling energy harvesting upon contact with external environments. In some cases, a single fiber generates electricity through interaction with the skin [74, 75, 76], while in other cases, it generates electricity through interaction with textiles. For example, Ma et al. controlled the material composition of fibers and the corresponding type of textiles, observing variations in peak voltage (Figure 3e) [41]. Furthermore, they demonstrated the application of this system not only for emerging harvesting devices but also as contact sensors for wearable electronics (Figure 3f).

Piezoionic energy harvesting refers to the process of converting mechanical energy into electrical energy when a twisted CNT yarn is stretched in an ionic liquid. Within the ionic liquid, the twisted CNT yarn forms capacitance through an electrical double layer (EDL). When stretched, the effective surface area of contact between the ionic liquid and the twisted CNT yarn changes, leading to a variation in capacitance. The resulting charge flow is then harvested as electrical energy (Figure 3g) [42, 52]. This method was first reported in a study by Kim et al., where they conducted energy harvesting using simple CNT yarn and CNT/Pt multiplied yarn [77]. Later, Wang et al. introduced various techniques to enhance the performance of the piezoionic energy harvester [78], and Zhang et al. further explored plying methods for better performance [42]. Their research demonstrated the energy conversion efficiency of up to 17.4% with stretch. Notably, Zhang et al.’s study provided an in‐depth analysis of the plying technique (Figure 3h) and its practical application in wearable systems (Figure 3i), highlighting its significance. Additionally, some studies utilized the piezoionic energy harvesters for energy harvesting and storaging textile [79] and strain sensors for gastric electronics [80].

In addition to the representative mechanical energy harvesters mentioned above, hybrid mechanical energy harvesters have also been continuously reported. Liu et al. developed a textile‐based system by weaving piezoelectric fiber energy harvesters and triboelectric energy harvesters, enabling simultaneous piezoelectric and triboelectric energy harvesting [81]. Similarly, Yu et al. reported a photoelectric conversion device that integrates a photomechanical actuator with electrostatic charge storage [82]. Additionally, as another example utilizing the EDL of CNT for mechanical energy harvesting, Xu et al. reported a fluidic nanogenerator [83].

Energy harvesters that operate based on various mechanical energy sources, such as human movement, have significant advantages when developed in a fiber form, as this allows for direct integration into traditional clothing. However, the number of harvesting methods that can be fabricated in fiber form is limited, as reviewed in this paper. Nevertheless, the abovementioned approaches have demonstrated effective energy harvesting from various bodily movements. Since different types of energy harvesters require different types of mechanical energy, future systems should not rely on a single type of energy harvester. Instead, combining multiple types of energy harvesters based on movement could maximize overall efficiency.

### 3.2 Thermal Energy

Thermal energy is ubiquitous in our daily lives, primarily as a byproduct of various energy conversion processes. For example, automobiles convert the chemical energy stored in gasoline into mechanical energy through combustion, generating and dumping significant amounts of heat as a byproduct through the exhaust pipe. From an energy conversion perspective, this heat represents a loss since it is dissipated into the surroundings without performing useful work. Similarly, heat is produced during battery charging, steel machining, incinerator operation, and even during respiration. Consequently, harvesting thermal energy is important given its widespread availability. To address this, the following subsections will discuss the representative mechanisms for fiber‐based thermal energy harvesting.

**FIGURE 4:**
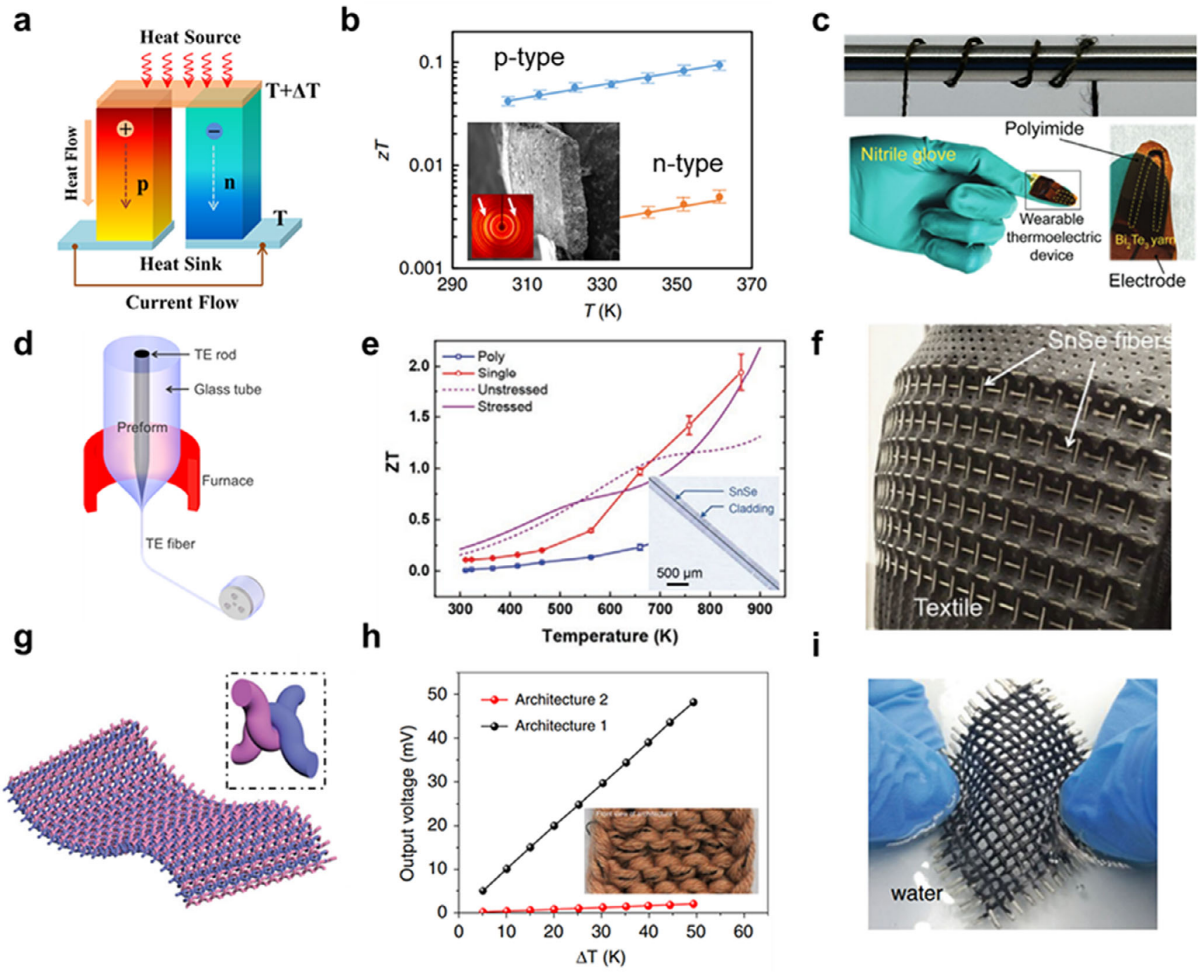
Fiber‐based thermal energy harvesters. (a) Schematic illustration of thermoelectric power generation using p‐ and n‐type semiconductors under a temperature gradient. Reproduced with permission from Ref. [53], Copyright 2023, The Authors, MDPI (b) *zT* values of 3D‐extruded Bi_2_Te_3_ fiber. Inset is the SEM image and XRD pattern of the fiber [43]. Reproduced under the terms of the CC‐BY 4.0 license, Copyright 2019, Springer Nature (c) Digital photograph of the all‐inorganic Bi_2_Te_3_ thermoelectric yarn and device. Reproduced with permission from Ref. [35], Copyright 2024 Wiley‐VCH GmbH. (d) Schematic showing the fabrication of the thermoelectric fiber using the molten core method. Reproduced with permission from Ref. [102], Copyright 2017, Elsevier Ltd. (e) *zT* values of SnSe fibers before (blue) and after (red) recrystallization process. Reproduced with permission from Ref. [44], Copyright 2020 Wiley‐VCH GmbH. (f) Digital photograph showing SnSe fibers woven in the textile. (g) Schematic drawing of CNT fibers with multiple pn junctions. Inset is the magnified image of the knot [111]. Reproduced under the terms of the CC‐BY 4.0 license, Copyright 2020, Springer Nature. (h) Output voltage of the CNT fiber woven in the textile [112]. Reproduced under the terms of the CC‐BY 4.0 license, Copyright 2020, Springer Nature. (i) Digital photograph of washing a CNT thermoelectric device in water [111]. Reproduced under the terms of the CC‐BY 4.0 license, Copyright 2020, Springer Nature.

Converting heat into a practical form of energy can be achieved using thermoelectric and pyroelectric phenomena. Pyroelectricic energy harvesters are typically fabricated as two‐dimensional fabrics, as they require a large surface area to be exposed to the thermal fluctuations [84]. In this review, therefore, we will focus on the thermoelectric fiber energy harvesters, which generate an electrical potential when a material is subjected to a temperature gradient. This phenomenon, known as the Seebeck effect, can be expressed mathematically as *V *= *S*Δ*T*, where *S* is the Seebeck coefficient and Δ*T* is the temperature difference between the hot and cold junctions [85]. The sign of S depends on the polarity of the majority carriers (negative for electrons, positive for holes) [86]. When n‐ and p‐type semiconductors are connected electrically in series and thermally in parallel (as illustrated in Figure 4a), the overall electrical potential is the sum of the voltages generated by the individual segments [53, 87]. Increasing the number of p–n junction pairs directly raises the output voltage, necessitating multiple pairs in thermoelectric generators to achieve high voltage under a given temperature gradient [88].

Commercial thermoelectric materials are typically metal chalcogenides, such as bismuth telluride (Bi_2_Te_3_) [89, 90, 91], lead telluride (PbTe) [92, 93, 94], or germanium telluride (GeTe) [95, 96]. These materials are extremely brittle and fragile, making it challenging to process them into complex shapes [97, 98]. As a result, they are usually fabricated as cuboids supported by rigid, planar ceramic substrates such as alumina (Al_2_​O_3_​) [99]. Such planar substrates, however, are not effective at absorbing heat from curved and soft surfaces like the human body or exhaust pipes. In this context, shape‐conformable thermoelectric fibers have the potential to significantly expand energy harvesting capabilities compared to conventional thermoelectric generators.

Peng et al. demonstrated that the extreme brittleness of thermoelectric materials can be alleviated through the use of an extruded thermoelectric composite (Figure 4b) [43]. In their approach, n‐ and p‐type Bi_2_Te_3_​ particles were dispersed in an appropriate solvent (e.g., dichloromethane) and combined with a non‐conductive polylactide‐*co*‐glycolide binder. Utilizing a 3D‐extrusion technique combined with roller compression, they fabricated Bi_2_Te_3_ ribbons with a volume fill fraction of 65% and a Herman's orientation parameter of 30% (inset in Figure 4b). The dimensionless thermoelectric figure‐of‐merit (*zT*)—which is directly proportional to the energy conversion efficiency—was measured as 0.005 for n‐type threads and 0.08 for p‐type threads. Although these values are somewhat lower than those of bulk materials, the thermoelectric threads demonstrated excellent flexibility, withstanding bending radii of less than 2 mm.

However, the use of non‐conducting polymer binders poses a critical obstacle to achieving high thermoelectric performance, as they significantly reduce electrical conductivity and increase the thermal resistance of the module [100]. Jang et al. addressed this issue by fabricating an all‐inorganic yet flexible thermoelectric Bi_2_Te_3_​ yarn (Figure 4c) [35]. Inspired by the flexibility of ultra‐thin nanoribbons, they first deposited Bi and Te onto a line‐patterned polyurethane acrylate (PUA) mold with 800‐nm‐wide features. Subsequent etching of the PUA mold using O_2​_ and CF_4_ plasma facilitated the detachment of the Bi and Te nanoribbons, which were then twisted into a yarn structure and thermally annealed to form the Bi_2_Te_3_​ phase with minimal secondary phase formation. The resulting all‐inorganic yarn exhibited improved electrical conductivity compared to composite yarns with binders and maintained performance over a wide temperature range where polymer binders typically become vulnerable. Moreover, the yarn demonstrated stable electrical resistance under harsh bending curvatures (0.5 mm^−1^) and tensile strains of approximately 5% over more than 1000 cycles.

Alternatively, a core‐and‐cladding structure offers an effective strategy for achieving flexibility without the need for binders or nanostructuring. As depicted in Figure 4d, bulk or powdered thermoelectric materials are melted at high temperatures and directly constrained by a glass cladding—a process known as the molten core method (MCM) [101], commonly used for fabricating glass fibers in optical communications. The main advantage of this method is that the resulting fiber can be flexible even if the core material is brittle, as the reduced thickness and diameter of the glass cladding lower the flexural rigidity sufficiently for free deformation. Using this concept, Zhang et al. fabricated flexible p‐type Bi_0.5_Sb_1.5_Te_3_ and n‐type Bi_2_Se_3_ core fibers with a diameter ranging from 50 to 1000 µm [102]. They found that the minimum bending radius varied significantly with fiber diameter, and they successfully constructed a 5‐pair thermoelectric device by wrapping the fiber around a water pipe with a 60‐mm width. Similarly, Sun et al. demonstrated polycrystalline Bi_2_Te_3_ core fibers with moderate flexibility, finding that texturing the Bi_2_Te_3_ core to exhibit a preferred orientation along the (0015) plane could more than double the *zT* values [103].

Unlike conventional thermoelectric materials, tin selenide (SnSe) exhibits remarkable thermoelectric properties often in its single‐crystal form but shows degraded performance in polycrystals [104, 105, 106]. Since the MCM process involves rapid melting and solidification of the core material within a glass container, the resulting SnSe typically crystallizes in a polycrystalline form. Zhang et al. reported that the *zT* value of polycrystalline SnSe fibers was only 0.58 at 862 K, significantly lower than that of the single‐crystal SnSe (Figure 4e) [44]. To overcome this limitation, they employed a CO_2_​ laser to recrystallize the as‐fabricated SnSe core, demonstrating that single‐crystal SnSe could be obtained at a laser velocity of 0.1 mm/s. The single‐crystal SnSe exhibited a *zT* value of approximately 2 at 862 K—nearly four times higher than its polycrystalline counterpart. Mechanical testing of 400‐µm‐thick SnSe fibers showed that they could withstand deflections of up to approximately 5 mm without significant failure, providing sufficient flexibility for integration into textiles (Figure 4f).

**FIGURE 5:**
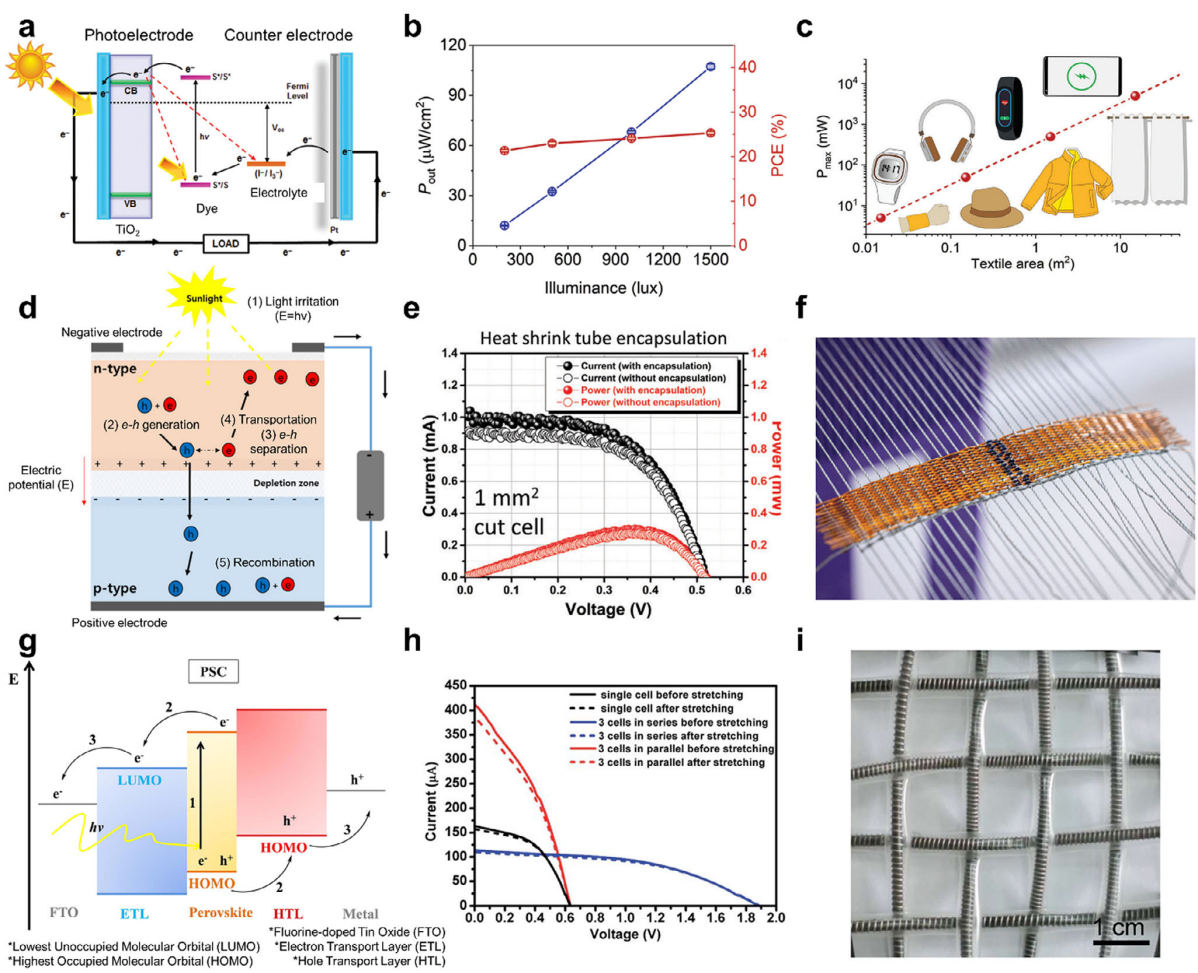
Fiber‐based optical energy harvesters. (a) Schematic of dye‐sensitized solar cells (DSSCs), illustrating electron excitation and charge transport. Reproduced with permission [123]. Copyright 2015 The Authors. Published by WILEY‐VCH Verlag GmbH & Co. KGaA, Weinheim. (b) Power density and efficiency of DSSC fibers under various illumination conditions. Reproduced with permission [46]. Copyright 2023 Wiley‐VCH GmbH. (c) Integration of DSSC fibers into textiles for wearable electronics. Reproduced with permission [46]. Copyright 2023 Wiley‐VCH GmbH. (d) Working principle of semiconductor photovoltaics (SPVs), showing electron–hole pair generation and separation. Reproduced with permission [49]. Copyright 2021 by the authors. Licensee MDPI, Basel, Switzerland. (e) Encapsulation effect on the performance of flexible crystalline silicon photovoltaic fibers. Reproduced with permission [47]. Copyright 2024 Wiley‐VCH GmbH. (f) Photographic representation of fiber‐based photovoltaic textiles. Reproduced with permission [47]. Copyright 2024 Wiley‐VCH GmbH. (g) Energy band diagram of perovskite solar cells (PSCs), highlighting charge generation and transport. Reproduced with permission [50]. Copyright 2021 The Author(s). Published by Elsevier Ltd. (h) Electrical performance of PSC fibers under different configurations before and after stretching. Reproduced with permission [48]. Copyright 2015 Royal Society of Chemistry. (i) Woven PSC fibers demonstrating flexibility and textile integration. Reproduced with permission [48]. Copyright 2015 Royal Society of Chemistry.

In addition to metal chalcogenide thermoelectrics, CNT yarns have attracted significant attention as fiber thermoelectric materials due to their high electrical conductivity, moderate Seebeck coefficient, and high specific strength [107, 108, 109]. Furthermore, the electrical conductivity and Seebeck coefficient of CNT yarns can be easily tuned by incorporating appropriate organic molecules [45, 110]. This facile doping process enables the straightforward formation of p–n junctions along the CNT yarn, enhancing the scalability and processability of CNT yarn‐based thermoelectric generators (Figure 5d). For example, Ding et al. developed an alternating extrude‐segment process to continuously form n‐ and p‐type single‐walled CNTs (SWCNTs) [111]. While typical SWCNTs exhibit p‐type conductivity, incorporating polyethyleneimine (PEI) converts them to n‐type. By alternately filling n‐ and p‐type SWCNT gels into a Teflon tube, they produced numerous p–n junction pairs within a single tube.

Similarly, Sun et al. utilized CNT fibers as a building block for fabricating thermoelectric fibers. In their approach, poly(3,4‐ethylenedioxythiophene): poly(styrenesulfonate) (PEDOT: PSS) was used as the p‐type hybridizing agent, while oleamine served as the n‐type dopant [112]. Employing an electrospraying technique, they selectively converted regions of the CNT fibers to either p‐type or n‐type, with the undoped regions acting as electrodes. These CNT fibers were directly woven into stretchable textiles, generating an open‐circuit voltage of approximately 50 mV under a temperature difference of 50 K (Figure 4h). Moreover, these fibers exhibited robust mechanical performance and were even washable, essential attributes for integration into practical garments (Figure 4i). These advancements in fiber thermoelectric energy harvesters hold great promise for self‐powered electronics and wearable devices even under severe deformation or extreme conditions such as water immersion. Aside from energy harvesting, it can be also utilized for monitoring temperature or heat in tactile sensors or robots, expanding applications of fiber thermoelectrics.

### 3.3 Optical Energy

Fiber optical energy harvesters convert light energy into electrical energy and hold significant potential in wearable and portable electronic applications [113, 114, 115]. Unlike mechanical or thermal energy harvesters that rely on external movement or heat sources, optical energy harvesters can continuously capture abundant sunlight outdoors or indoor lighting, providing a virtually unlimited energy supply. Integrating these devices into textiles is particularly advantageous since humans naturally operate in well‐lit environments, making energy harvesting seamless [116]. To provide a detailed overview of this technology, the following subsections will explore the three primary categories of fiber‐based photovoltaics: dye‐sensitized solar cells (DSSCs) [117, 118, 119, 120], semiconductor photovoltaics (SPVs) [47, 49], and PSCs [121, 122].

DSSCs operate by using dye molecules to absorb photons, exciting electrons to a higher energy state (Figure 5a) [123, 124, 125]. These electrons are then injected into the conduction band of a semiconductor such as titanium dioxide (TiO_2_) and subsequently collected by an electrode, generating an electric current. The oxidized dye is regenerated via a redox reaction in the electrolyte, typically containing iodide/triiodide pairs, allowing continuous operation. DSSCs are particularly effective in low‐light conditions, such as indoor environments, due to their strong absorption in the visible light range (400–700 nm). For example, Zhu et al. developed a novel dye‐sensitized indoor photovoltaic fiber (IPVF) with a certified power conversion efficiency (PCE) of 25.53% under 1500 lux illumination, as shown in Figure 5b [46]. This efficiency was achieved by utilizing a hybrid TiO_2_ structure to enhance light absorption and charge transport, combined with a core‐sheath CNT counter electrode. The hybrid TiO_2_ layer ensured stable interfaces and minimized charge recombination, leading to efficient charge transport. Additionally, the IPVFs demonstrated over 95% performance retention after 1000 mechanical deformation cycles, showcasing their durability for wearable applications. These fibers can be woven into textiles for wearable electronics, including self‐charging smart fabrics and portable communication devices (Figure 5c).

Semiconductor photovoltaics harvest energy by leveraging the photoelectric effect, where photons absorbed by the semiconductor material generate electron–hole pairs. In crystalline silicon photovoltaics, which operate over a broad spectral range (400–1100 nm), the built‐in electric field of the p–n junction separates the electron–hole pairs, directing electrons to the n‐side and holes to the p‐side (Figure 5d) [49]. These charges are collected by interdigitated back contacts, completing the circuit. As an example, Jin et al. demonstrated flexible crystalline silicon photovoltaic fibers with a PCE of 11% under air mass 1.5G illumination, as shown in Figure 5e [47]. These fibers were fabricated by integrating diced crystalline silicon cells onto flexible substrates and showed excellent durability, enduring up to 8000 bending cycles without significant degradation in performance. Encapsulation with ethylene vinyl acetate polymer was shown to enhance device stability and improve light absorption by reducing optical losses at the interfaces. Post‐encapsulation analysis demonstrated increased short‐circuit current density, contributing to the observed improvement in PCE. Their robustness and scalability make them ideal for wearable applications such as outdoor energy‐harvesting fabrics and portable power supplies (Figure 5f).

Perovskite solar cells operate by absorbing light over a wide spectral range (400–1200 nm) using perovskite materials, which generate excitons, or electron–hole pairs, upon light absorption (Figure 5g) [50]. These excitons dissociate at the interfaces between the perovskite layer and charge transport layers due to the built‐in electric field, facilitating efficient charge separation. The electrons are transported through electron transport layers such as TiO_2_ to the cathode, while holes are transferred to the anode via hole transport layers like spiro‐OMeTAD. Representative, Deng et al. introduced elastic perovskite solar cell fibers using spring‐shaped titanium wires coated with perovskite materials. These fibers maintained stable photovoltaic performance and mechanical resilience even after repeated stretching. Additionally, these PSC fibers could be connected in series to increase the output voltage from 0.63 to 1.88 V, or in parallel to enhance the output current from 156 to 412 µA, as shown in Figure 5h [48]. Such fibers, which can be woven into textiles, show great promise for wearable electronics (Figure 5i).

Each of these optical energy harvesting technologies has unique strengths and limitations. DSSCs are particularly suited for low‐light indoor applications, but they require improvements in electrolyte stability and broader spectral absorption. Semiconductor photovoltaics excel under strong sunlight and provide high durability, though their flexibility and low‐light performance need enhancement. Perovskite solar cells offer high efficiency across diverse lighting conditions, but face challenges related to environmental stability and long‐term durability. Advancements in these technologies will enable transformative applications in wearable electronics and sustainable energy systems, making fiber‐optical energy harvesters a crucial component in the future of energy innovation [126, 127, 128, 129, 130].

## 4 Conclusions and Outlook

In this review, we have systematically examined the recent progress in fiber energy harvesters, highlighting their unique advantages over conventional bulk and thin‐film devices.

To summarize, Section 2 introduced fabrication techniques for the formation of functional fibers by classifying them into two: bottom–up and top–down approaches. Bottom–up approaches—such as electrospinning, chemical vapor deposition, and electron beam deposition—build fibers from nanoscale building blocks and enable the formation of highly stretchable, multifunctional yarns. In contrast, top–down methods, including dip coating and spray coating, offer the versatility of functionalizing commercially available fibers, despite some limitations regarding single‐material fabrication. Additional processing techniques, such as twisting (single‐ and multiplying) and cable or core‐spun plying, were also highlighted for their ability to further enhance the mechanical and functional properties of fiber assemblies. Then, Section 3 categorized fiber energy harvesters based on their primary energy sources—mechanical, thermal, and optical. For mechanical energy harvesters, diverse mechanisms including piezoelectric, triboelectric, and piezoionic systems were introduced, demonstrating how fiber architectures can efficiently convert human motion into electrical energy. For thermal energy harvesting, we examined thermoelectric fibers that exploit the Seebeck effect to generate electric power from temperature gradients, along with approaches that address the inherent brittleness of conventional thermoelectric materials through composite structures, core‐cladding techniques, and CNT yarns. Finally, optical energy harvesters including dye‐sensitized, semiconductor, and perovskite‐based photovoltaic fibers were reviewed, which show great promise for indoor and outdoor applications due to their ability to capture ambient light.

However, fiber energy harvesters face several challenges and require further improvements to be competitive in the energy harvesting field. First, the relatively low energy conversion efficiency of fiber energy harvesters compared to their bulk counterparts must be addressed. This may stem from the degradation of electrical conductivity in fiber materials during processing. For example, while bulk Bi_2_Te_3_ exhibits an electrical conductivity of approximately 10^3^ S cm^−^^1^ when properly doped, the 3D‐extruded Bi_2_Te_3_ composite reaches at most ∼10 S cm^−^^1^. Similarly, fiber perovskite solar cells demonstrate a PCE of only about 5.2%, in contrast to roughly 26% for their bulk counterparts. These efficiency gaps lead to poor energy harvesting performance, particularly when coupled with the limited availability of the primary energy source. In addition, interfacial losses, carrier recombination, and morphological defects introduced during fiber fabrication further exacerbate performance limitations. Addressing these issues requires collective innovations in material synthesis, interface engineering, and device architecture tailored specifically for fiber geometries. Moreover, the total energy output of fiber energy harvesters is difficult to generalize, as it strongly depends on the available input energy and the effective harvesting area when fibers are integrated into garments. Under typical wearable conditions, the generated electrical power is generally expected to be in the µW–mW range, and achieving continuous W‐level power at the garment scale remains challenging.

Second, the biocompatibility and safety of fiber energy harvesters must be considered for wearable electronics. Many energy harvesting materials exhibit a degree of toxicity when in contact with humans. For instance, the International Agency for Research on Cancer classifies CNTs as possible carcinogens, which may induce fibrosis, oxidative stress, and inflammatory responses in the respiratory system. Metal chalcogenides, perovskites, and even some polymeric fibers can also be harmful, necessitating stringent safety measures for their integration into everyday life. This includes the use of encapsulation layers, surface passivation, or the development of intrinsically biocompatible alternatives. Long‐term exposure studies and rigorous toxicological assessments are also essential for certifying materials for safe use in on‐skin or near‐skin applications.

Third, the stability of fiber energy harvesters in aqueous environments is critical. Since most garments are exposed to aqueous environments during washing and daily use, fiber‐based devices must withstand repeated exposure to water, high humidity, and temperature fluctuations without degradation in energy harvesting performance. However, many fiber‐based devices suffer from delamination, corrosion, or loss of conductivity when exposed to water and detergents. Encapsulation strategies using hydrophobic polymers, fluorinated coatings, or self‐healing materials have shown promise, but often introduce trade‐offs in flexibility or breathability. Importantly, the encapsulation material must be tailored to the type of energy harvester: soft, low‐modulus layers for mechanical harvesters; thermally conductive, low‐capacitance materials for thermal harvesters; and optically transparent materials for optical harvesters. Therefore, the development of robust, wash‐resistant architectures that preserve device performance without compromising wearability remains a key research priority.

Last but not least, the production cost of fiber energy harvesters needs to be significantly reduced to compete with conventional commercial fibers used in textiles and electronics. Current fabrication processes—such as electrospinning, chemical vapor deposition, or electron beam deposition—often involve precise control over processing parameters, cleanroom environments, and high‐vacuum or high‐temperature systems, all of which contribute to high capital and operational costs. In addition, the use of costly precursor materials, such as noble metals or rare elements, further elevates manufacturing expenses. Batch‐to‐batch variability and low throughput also hinder economic scalability. Therefore, developing scalable, high‐throughput production techniques based on roll‐to‐roll printing, solution‐based fiber drawing, or 3D fiber extrusion is essential for lowering overall production costs while maintaining performance and material integrity. Integration with existing textile manufacturing infrastructure will also be critical to facilitate large‐scale adoption.

Despite these challenges, fiber energy harvesters hold great promise for advancing wearable electronics in everyday life. Extensive interdisciplinary research is required to overcome these limitations. For instance, in materials science, the development of lead‐free perovskites or intrinsically stretchable organic conductors beyond PEDOT: PSS could address toxicity and mechanical failure issues. Simultaneously, structural innovations in mechanical engineering, such as kirigami or serpentine fiber architectures, can significantly enhance deformability without compromising electrical performance. Furthermore, integrating machine learning techniques for high‐throughput material screening and lifetime prediction may accelerate the discovery of optimal fiber compositions and morphologies. Strategies such as passivating fiber energy harvesters with biocompatible and washable components (e.g., poly(dimethylsiloxane) [PDMS]) or developing safe, non‐toxic devices that remain stable in water offer potential solutions. Moreover, fully automated fabrication using robotic manufacturing or innovative, low‐cost, solution‐based processes for kilogram‐scale production could further reduce costs. In the future, we can expect clothing to be made from these energy‐harvesting materials, serving as dominant power sources in our daily lives.

## Conflicts of Interest

The authors declare no conflicts of interest.

## Data Availability

No new data were created or analyzed in this study. Data sharing is not applicable to this article.
